# Relationship Between Curative Effect and Serum Inflammatory Factors Level in Male Patients With First-Episode Schizophrenia Treated With Olanzapine

**DOI:** 10.3389/fpsyt.2021.782289

**Published:** 2021-12-09

**Authors:** Jun Ma, Yanting Zhang, Zhuowei Huang, Xuebing Liu, Luxian Lv, Yi Li

**Affiliations:** ^1^Affiliated Wuhan Mental Health Center, Tongji Medical College of Huazhong University of Science & Technology, Wuhan, China; ^2^Suzhou Guangji Hospital, Suzhou, China; ^3^Henan Key Lab of Biological Psychiatry, Xinxiang Medical University, Xinxiang, China

**Keywords:** schizophrenia, curative effect, hs-CRP, IL-1β, IL-6, IL-17, TGF-β1

## Abstract

**Background:** A growing body of evidence shows that immune system disorders are one of the important etiological factors of schizophrenia. Inflammatory cytokines play a very critical role in the pathogenesis and treatment of schizophrenia. However, in the actual clinical practice, there is still a lack of confirmed biological indicators that can be used to evaluate the therapeutic effect of antipsychotics.

**Methods:** In this study, 82 male patients with first-episode schizophrenia and 30 healthy controls were included. The Positive and Negative Syndrome Scale (PANSS) scores were evaluated, and the serum levels of high-sensitivity C-reactive protein (hs-CRP), interleukin 1β (IL-1β), interleukin 6 (IL-6), interleukin 17 (IL-17), and transforming growth factor β1 (TGF-β1) were detected, both at baseline and 4 weeks later. The patients were divided into two groups, the effective group and the ineffective group, according to the reduction rate of PANSS.

**Results:** In the case group, the levels of hs-CRP were significantly elevated (*p* = 0.00), whereas IL-1β, IL-6, and IL-17 were significantly reduced as compared to the baseline (*p* = 0.01, 0.02, and 0.00, respectively). Importantly, the baseline levels of the five inflammatory factors were significantly higher in the case group as compared to the control group (*p* = 0.00, 0.00, 0.00, 0.00, and 0.00, respectively). Post-treatment, the serum levels for IL-1β, IL-6, and IL-17 were significantly higher in the effective group than in the ineffective group (*p* = 0.00, 0.00, and 0.01, respectively). For every increase in the amount of IL-1β, the risk of ineffectiveness increased by 7% (OR = 0.93 [0.86–1.00]; *p* = 0.04), whereas for every increase in the amount of IL-17, the risk of ineffectiveness increased by 5% (OR = 0.95 [0.90–0.99]; *p* = 0.03).

**Conclusion:** The results of the study showed that the levels of inflammatory factors in patients with different therapeutic effects were different, and the changes in the amounts of IL-1β and IL-17 acted as predictors of poor efficacy.

## Introduction

In the past few decades, significant evidence established the idea that the complex interaction between the immune-inflammatory system and the brain might have important etiological and therapeutic significance with regard to neuropsychiatric diseases (1). In fact, several studies reported that inflammatory markers, such as inflammatory/pro-inflammatory cytokines, are one of the etiological factors involved in a variety of mental disorders (2–4). A meta-analysis of the levels of inflammatory factors present in the cerebrospinal fluid reported increased levels of pro-inflammatory factors in the cerebrospinal fluid of patients with schizophrenia, whereas the levels of anti-inflammatory factors were found to be reduced (5). Similarly, significantly higher levels of some of the inflammatory factors in the peripheral nervous system were reported in patients with first-episode schizophrenia or relapses, as compared to healthy controls (6). Importantly, several studies have also provided the most convincing evidence for the inflammation theory of schizophrenia. For example, some of these studies involved the use of minocycline (an antibiotic) for the treatment of patients with schizophrenia, and improvement was observed in the cognitive symptoms and negative symptoms of the patients with reduction in the levels of some of the pro-inflammatory factors (7, 8). In parallel to this, antipsychotic drugs have also been shown to exhibit similar anti-inflammatory properties. For example, an animal study showed that atypical antipsychotics such as clozapine and olanzapine have the unique effect of inhibiting the levels of pro-inflammatory factors (9). Another example, *in vitro* assessment of second-generation antipsychotic drugs, reported a reduction in the mRNA expression levels for IL-1β, IL-6, and TNF-α in the peripheral blood of healthy people, which further resulted in a reduction in the concentration of these inflammatory factors (10). The reliability and persuasiveness of this conclusion have been further enhanced by the outcomes of clinical studies/research. In a meta-analysis, which included 23 studies (762 participants in total), antipsychotic medication was reported to significantly reduce the plasma levels of IL-1β and interferon γ (IFN-γ) in patients with schizophrenia (11). In another study, patients with schizophrenia benefited more from the combined use of anti-inflammatory and antipsychotic drugs than the use of antipsychotic drugs alone (12). Altogether, the occurrence of inflammatory state during psychosis and the accumulated evidence for anti-inflammatory effects of antipsychotic drugs support the use of inflammation as a potential new therapeutic target for the management of schizophrenia.

Currently, very limited data are available to support the relationship between inflammatory factor levels and psychopathology. In a study involving patients with chronic schizophrenia, Xiu et al. reported a positive correlation between interleukin 18 (IL-18) and the PANSS General Psychopathology scale score (13). Another study showed that an increase in interleukin 2 (IL-2) levels might be related to cognitive symptoms and positive symptoms of chronic schizophrenia (14). Although the theory of the central role of inflammation in schizophrenia and the anti-inflammatory effects of antipsychotics rely on and complement each other, it remains unknown whether there is a correlation between the level of inflammatory factors and the therapeutic effect. In other words, there are no exact biological indicators that can be applied for the evaluation of the efficacy of antipsychotic drugs. In order to answer the above mentioned questions, we assessed the effects of olanzapine on first- episode male schizophrenia patients and compared the levels of five inflammatory cytokines, namely high-sensitivity C-reactive protein (hs-CRP), interleukin 1β (IL-1β), interleukin 6 (IL-6), interleukin 17 (IL-17), and transforming growth factor β1 (TGF-β1), between two groups of the patients that demonstrated different curative effects in order to explore the potential biological indicators that can serve to determine the efficacy of these drugs in patients with schizophrenia.

## Methods

### Subjects

Patients hospitalized in the Second Affiliated Hospital of Xinxiang Medical College during the period from February 2015 to October 2016 were enrolled in this study. The following were the inclusion criteria for subject selection: (1) patients meeting the diagnostic criteria of schizophrenia as per the International Classification of Diseases 10th Revision (ICD-10), with a disease course of ≤ 60 months and no antipsychotic treatment or treatment time of <2 weeks; (2) Han nationality, age 15–60 years, male sex; (3) the Positive and Negative Syndrome Scale (PANSS) score of ≥60; (4) olanzapine use alone within 4 weeks of admission that was subsequently increased to the effective treatment amount within 2 weeks. The average daily dose of olanzapine was 10–20 mg. The exclusion criteria were as follows: severe physical diseases, immune diseases, acute (slow) infections, alcohol or drug dependence, intellectual disability, bipolar disorder, and other mental diseases, women, and children. The following were the withdrawal criteria: suffering from serious physical diseases during hospitalization; the combined use of other antipsychotics; and a serum hs-CRP level of >10 mg/L at any time, which was considered to indicate acute infection and withdrawal from the study.

During the same period, we recruited 30 healthy men as controls who were matched for age and years of education with the case group in the community.

This study was reviewed and approved by the ethics committee of the Second Affiliated Hospital of Xinxiang Medical College. All subject guardians were informed about the study and their signed informed consent were obtained.

### Research Design

This study was designed as a case–control study. A comparison of the differences between the baseline inflammatory factor levels between the case group and the healthy control group was performed. Comparison of the changes in the inflammatory factor levels before and after treatment with olanzapine in the included patients, the relationship between the serum hs-CRP, IL-1β, IL-6, IL-17, and TGF-β1 levels, and the efficacy were explored. The patients were scored on the PANSS scale on the day after admission, and the venous blood was drawn to determine the serum levels of hs-CRP, IL-1β, IL-6, IL-17, and TGF-β1. At the end of the 4th week of treatment, the PANSS scale was determined for the second time, and the serum hs-CRP, IL-1β, IL-6, IL-17, and TGF-β1 levels were re-measured. Binary logistic regression analyses were performed to construct a prediction model in order to determine the impact of general clinical data and changes in the level of inflammatory factors on the curative effect of patients.

### Therapeutic Method

The included patients were treated with olanzapine alone, and the drug dose was gradually adjusted according to the condition. The drug dose was added to the effective therapeutic dose (10–20 mg/day) within 2 weeks. During the treatment, benhexol tablets (2–6 mg/day) and propranolol tablets (10–30 mg/day) were used to avoid adverse drug reactions, and benzodiazepines were used in combination to improve the sleep quality. Antidepressants, emotional stabilizers, and other drugs were not used in combination, nor other antipsychotics were used in combination; modified electroconvulsive (MECT) therapy was also not used.

### Determination of Serum Inflammatory Factors

Fasting elbow venous blood (5 ml) was collected on the day of admission and at 07:00–08:00 a.m. of the fourth weekend of treatment. The blood sample was placed in a glass tube and agglutinated at room temperature and centrifuged at 4°C at 3,000 rpm for 10 min. The upper serum layer was separated and stored at −80°C for later use. After the samples were collected, the levels of hs-CRP, IL-1β, IL-6, IL-17, and TGF-β1 were measured by enzyme-linked immunoassay (ELISA). All kits were provided by Wuhan Bode Bioengineering Co., Ltd., and the operation process was performed in strict accordance with the manufacturer's instructions. The sampling time, method, storage, and cytokine detection of the blood samples in the healthy control group were conducted in the same manner as for the case group.

### PANSS Score Reduction Rate and Grouping

Three professionally trained psychiatrists scored the included patients with the PANSS scale on the day of admission and at the end of 4 weeks of treatment to ensure consistency in the scoring results. The clinical treatment effect was evaluated by the PANSS score reduction rate (%) = (baseline score – post-treatment score)/(baseline score – 30) × 100%, and the patients were finally divided into the PANSS reduction rate < 50% group (ineffective group, labeled as group A) and the PANSS reduction rate ≥ 50% group (effective group, labeled as group B).

### Data Analysis

The normally distributed continuous measurement data obtained were expressed as the mean and standard deviation, and the categorical variables were counted. Paired *t*-test was performed to compare the same group of data before and after, and an independent sample *t*-test was used to compare the data of different groups. Binary logistic regression was employed to construct a model in order to evaluate the influencing factors of the curative effect. The significance level of all statistical tests was set to *p* < 0.05 (two tails). Data analysis was performed using IBM SPSS version 26.0.

## Results

### General Characteristics of Clinical Treatment

A total of 111 individuals completed the entire course of the study. During the treatment, 12 cases additionally received antidepressants, 4 cases additionally received valproate, and 13 cases for serum hs-CRP levels > 10 mg/L, at least once during the assessment, were eliminated from the study. Toward the end of the study, 82 cases were screened and included in statistical analyses. The clinical characteristics of the patients included in the study are shown in Table 1.

**Table 1:** The general clinical characteristics of the included patients.

**Index**	**Included patients (*n* = 82)**
Age—years	
Mean (SD)	30.86 (9.96)
Range	15–59
The course of schizophrenia**—**years (SD)	2.95 (1.61)
Years of Education**—**years	5.62 (1.04)
Duration of treatment-naïve**—**months	20.00 (9.01)
Positive family history**—***n* (%)	17 (20.70%)
Prescription benhexol**—***n* (%)	2 (2.43%)
Prescription propranolol**—***n* (%)	7 (8.54%)
Prescription benzodiazepine**—***n* (%)	26 (31.71%)
Marital status**—***n* (%)	
Unmarried	34 (41.50%)
Having a spouse	41 (50.00%)
Divorced	0 (0)
Others	7 (8.5%)

### PANSS Score Reduction Rate and Grouping Post-treatment

After 4 weeks of olanzapine treatment, the patients were divided into two groups as per the reduction rate of PANSS score. The ineffective group (group A) was characterized by a score reduction rate of <50%, and the effective group (group B) exhibited a score reduction rate of ≥50% (Table 2).

**Table 2:** PANSS score reduction rate and grouping after 4 weeks of treatment.

	***N***	**Baseline**	**Post-treatment**	**Reduction rate (%)**
Total score	82	89.34 ± 11.50	67.15 ± 18.68	38.84 ± 25.33
Group A	42	92.4 ± 11.23	82.38 ± 12.74	16.38 ± 11.75
Group B	40	86.05 ± 10.96	51.15 ± 6.51	62.43 ± 8.56

*Group A: ineffective group, the score reduction rate of PANSS <50%; Group B: effective group, the score reduction rate of PANSS ≥50%*.

### Changes in the Levels of Five Target Inflammatory Factors, Before and After Treatment

In the case group, the levels of hs-CRP were found to be significantly elevated as compared to the baseline (*p* = 0.00). In comparison to this, IL-1β, IL-6, and IL-17 levels were significantly reduced, and the differences were statistically significant (*p* = 0.01, 0.02, and 0.00, respectively). The baseline levels of the five inflammatory factors were significantly higher in the case group as compared to healthy controls, and the differences were statistically significant (*p* = 0.00, 0.00, 0.00, 0.00, and 0.00, respectively). Post-treatment, the levels of hs-CRP, IL-1β, IL-6, and TGF-β_1_ were higher as compared to healthy controls, and the differences were statistically significant (*p* = 0.00, 0.00, 0.00, and 0.00, respectively) (Table 3).

**Table 3:** Changes of five inflammatory factors before and after treatment.

	**Baseline**	**Post-treatment**	**Healthy control**
hs-CRP (mg/L)	0.81 ± 0.95	1.94 ± 1.85^a^	0.25 ± 0.46^a^^b^
IL-1β (ng/L)	45.94 ± 13.01	36.56 ± 12.28^a^	29.10 ± 11.23^a^^b^
IL-6 (ng/L)	50.57 ± 13.68	38.62 ± 14.68^a^	30.87 ± 8.46^a^^b^
IL-17 (ng/L)	45.64 ± 13.73	36.51 ± 14.10^a^	35.26 ± 10.98^a^
TGF-β_1_ (ng/L)	47.94 ± 9.00	45.41 ± 19.24	35.98 ± 15.36^a^^b^

a *p < 0.05 compared with the baseline value*.

b *p < 0.05 compared with the post-treatment value*.

### Differences in the Levels of Five Target Inflammatory Factors in Different Groups and at Different Periods

Post-treatment, the serum levels of hs-CRP in patients belonging to groups A and B increased significantly as compared to the baseline, and the differences were statistically significant (*p* = 0.0 and 0.00, respectively). In comparison to this, IL-1β, IL-6, and IL-17 levels were recorded to be significantly lower in groups A and B as compared to the baseline, and the differences were statistically significant (A: *p* = 0.00, 0.02, and 0.00, and B: *p* = 0.01, 0.02, and 0.02, respectively). Post-treatment, the serum levels of IL-1β, IL-6, and IL-17 were recorded to be significantly higher in group B as compared to group A, and the differences were statistically significant (*p* = 0.00, 0.00, and 0.01, respectively) (Table 4).

**Table 4:** Comparison of the levels of five inflammatory factors between groups A and B before and after treatment.

	***n***	**hs-CRP (mg/L)**	**IL-1β (ng/L)**	**IL-6 (ng/L)**	**IL-17 (ng/L)**	**TGF-β_1_ (ng/L)**
Group A	42					
Baseline		0.90 ± 1.14	45.54 ± 13.69	51.14 ± 11.77	44.15 ± 14.54	48.20 ± 9.54
Post-treatment		2.12 ± 2.15^a^	33.93 ± 13.36^a^	35.29 ± 12.42^a^	30.68 ± 12.81^a^	47.05 ± 20.50
Group B	40					
Baseline		0.72 ± 0.71	46.36 ± 12.41	49.96 ± 15.56	47.21 ± 12.81	47.67 ± 8.49
Post-treatment		1.75 ± 1.49^a^	39.32 ± 10.47^a^^b^	42.13 ± 16.16^a^^b^	42.62 ± 12.84^a^^b^	43.67 ± 17.92^a^

a *p < 0.05 compared with the baseline value*.

b *p < 0.05 compared with group A in the same period*.

### Logistic Regression Model for the Identification of Predictive Factors of Efficacy in Patients, After 4 Weeks of Treatment

A binary logistic regression model was used to analyze the factors influencing efficacy. Herein, efficacy (marked PANSS score reduction rate < 50% was considered ineffective, and marked PANSS score reduction rate ≥ 50% was considered effective) was used as the dependent variable, and age, course of schizophrenia, years of education, duration of treatment-naïve, family history, marital status, and the amount of change in five target inflammatory factors were used as covariates. The prediction model was found to be statistically significant (*B* = −0.05; *p* = 0.02; OR = 0.95). The prediction accuracy rate was recorded to be 74.4%. For every increase in the amount of IL-1β, the risk of ineffectiveness increased by 7% (OR = 0.93 [0.86–1.00]; *p* = 0.04); and for every increase in the amount of IL-17, the risk of ineffectiveness increased by 5% (OR = 0.95 [0.90–0.99]; *p* = 0.03) (Table 5).

**Table 5:** Factors predictive of efficacy in patients after 4 weeks of treatment: logistic regression model.

	**B (SE)**	**OR**	**95% CI**	***p***
Constant	0.53 (1.96)	1.69		0.79
Age	0.16 (0.03)	1.02	0.96–1.08	0.59
Course of schizophrenia	0.19 (0.23)	1.21	0.77–1.92	0.41
Years of education	0.15 (0.27)	1.17	0.67–2.04	0.59
Duration of treatment-naïve	−0.04 (0.05)	0.97	0.88–1.06	0.44
Family history	−0.56 (0.70)	0.57	0.15–2.24	0.42
Marital status	0.08 (0.62)	1.85	0.32–3.67	0.90
Δ hs-CRP	0.10 (0.19)	1.11	0.77–1.59	0.58
Δ IL-1β	−0.08 (0.04)	0.93	0.86–1.00	0.04^*^
Δ IL-6	−0.03 (0.22)	0.97	0.93–1.01	0.17
Δ IL-17	−0.05 (0.25)	0.95	0.90–0.99	0.03^*^
Δ TGF-β_1_	0.01 (0.17)	1.01	0.97–1.04	0.70

*SE, standard error; Δ, baseline level—post-treatment level*;

* *p < 0.05*.

## Discussion

The present study confirmed that patients with first-episode schizophrenia exhibited relatively higher levels of inflammatory factors, during the acute stage. After 4 weeks of treatment with the antipsychotic drug olanzapine, this inflammatory state was alleviated to a certain extent. Interestingly, it was observed that the levels of inflammatory factors were significantly higher in patients with good curative effect as compared to the patients with poor curative effect, which further showed that a negative correlation existed between the severity of inflammation and curative effect. However, hs-CRP seems to be independent of the above conclusions, as the trend for the change in this indicator was quite opposite to the others. Most importantly, the study identified that the amount of change in IL-1β and IL-17, before and after the treatment, could act as predictive factors for assessing treatment effects.

For a long time, it was considered that schizophrenia is a disease of “chronic mild central inflammation” (15). In fact, this theory has always been an important part of the etiological hypothesis of schizophrenia. Irrespective of the involvement of the kynurenine pathway (16) or the vulnerability-stress-information model (17), all these theories/studies support the idea of the existence of a causal relationship between inflammation and schizophrenia. Additionally, the pathway of inflammation leading to schizophrenia is interactive and complex. Altogether, all these studies suggest that an imbalance in the levels of inflammatory factors is directly related to the disease (1, 18, 19). In concordance with these reports, the present study shows that the levels of peripheral inflammatory cytokines were significantly higher in the acute phase of first-onset schizophrenia, as compared with healthy people. Post-treatment, a significant reduction in the levels of multiple cytokines was recorded. These results further supported the role of inflammation in the pathogenesis and treatment of schizophrenia.

In a study involving meta-analysis of 47 *in vivo* studies on schizophrenia, treatment of patients with antipsychotic drugs resulted in a significant reduction in the patients' peripheral serum levels for IL-1β and IL-6 (20), indicating that antipsychotics exhibited anti-inflammatory effects, and thus could efficiently balance the high-level inflammatory state of schizophrenic patients. This conclusion was further verified by the results of the present study, wherein a significant reduction was reported in the peripheral IL-1β, IL-6, and IL-17 levels of the patients after taking olanzapine for 4 weeks, regardless of the curative effect. The only contrast to this conclusion was the change in the level of hs-CRP, which was completely different from the change in the trend reported for the other four inflammatory factors. Although the present study insisted that antipsychotics reduced the inflammatory level of patients, a significant increase in the level of hs-CRP was recorded, toward the end of the study. In a clinical study involving 105 patients with schizophrenia, treatment of the patients with atypical antipsychotics was associated with increased levels of peripheral CRP in the patients (21). Another study conducted in India reported similar results (22). The results of the present study were consistent with the findings of the above two studies. However, this increase in hs-CRP levels seems to be short-lived, and this state would disappear after a longer period of antipsychotic treatment (23). The observed increase in hs-CRP levels might be attributed to antipsychotic drugs that mediated hindrance in the formation of hs-CRP complex, which increased the content of free hs-CRP, while reducing the damage of hs-CRP to nerve cells (24). Therefore, it was believed that although the trend of change was different, that is, the level of hs-CRP increased, the level of the other three inflammatory factors (IL-1β, IL-6, and IL-17) decreased in such a way that it ensured a decrease in inflammatory titer and avoided further damage to the central nervous system, assuring that the patients would eventually benefit.

In addition to this, the present study compared the differences in the levels of five inflammatory cytokines in the two groups of patients with different curative effects and reported that the serum levels of IL-1β, IL-6, and IL-17 were higher in patients with good curative effects. Although correlation analysis was not performed, these results suggested that the efficacy was positively correlated with the titer of inflammatory factors at post-treatment, to some extent. Thus, it is suggested that on the premise of the absence of difference in the level of inflammatory factors in the baseline, the greater the decrease in the level of inflammatory factors after treatment, the lower the inflammatory titer in the patient post-treatment, but the worse will be the curative effect. This seems to be contrary to the usual understanding regarding “inflammatory diseases.” In a previous study, Dimitrov et al. showed that the levels of a variety of inflammatory cytokines, including IL-1β and IL-6, were positively correlated with the PANSS score (25). Another longitudinal study involving the level of inflammatory factors and psychopathology of schizophrenia analyzed the relationship between the reduction of mental symptoms after treatment and the level of inflammatory factors in the baseline period. The results of the study showed that the level of IL-6 in the baseline period was related to the reduction of PANSS score after treatment (26). Although both these studies combined the levels of inflammatory factors with psychopathology, the differences in the levels of inflammatory factors in groups with different PANSS score reduction rates were not analyzed, which represents the novelty of the present study. No previous studies have been reported so far to support or oppose the present study. In order to further explain the relationship between the therapeutic effect and the changes in the levels of inflammatory factors, a binary logistic regression analysis was performed, and potential factors predicting the therapeutic effect of patients were evaluated. In particular, the amount of change in IL-1β and IL-17 was identified as independent predictors of efficacy.

In conclusion, the findings of the present study support the idea that schizophrenia patients are characterized by inherent immune disorders. Olanzapine could promote the reduction in the levels of inflammatory factors, and thus could correct this disorder. The levels of inflammatory factors in patients with different therapeutic effects were recorded to be different, and the amount of change in IL-1β and IL-17 was identified as predictors of poor efficacy.

## Data Availability Statement

The original contributions presented in the study are included in the article/supplementary material, further inquiries can be directed to the corresponding author/s.

## Ethics Statement

The studies involving human participants were reviewed and approved by the Ethics Committee of the Second Affiliated Hospital of Xinxiang Medical College. The patients/participants provided their written informed consent to participate in this study.

## Author Contributions

LL made substantial contributions to conception and design of the review. JM and YZ collected and arranged clinical data. JM and ZH drafted the manuscript. XL revised the manuscript critically for important intellectual content. ZH ensured that questions related to the accuracy or integrity of any part of the work were appropriately investigated and resolved. YL and LL gave final approval of the version to be published. All authors contributed to the article and approved the submitted version.

## Funding

This study was funded by the Wuhan Health Commission (WX19Y12, JM, PI).

## Conflict of Interest

The authors declare that the research was conducted in the absence of any commercial or financial relationships that could be construed as a potential conflict of interest.

## Publisher's Note

All claims expressed in this article are solely those of the authors and do not necessarily represent those of their affiliated organizations, or those of the publisher, the editors and the reviewers. Any product that may be evaluated in this article, or claim that may be made by its manufacturer, is not guaranteed or endorsed by the publisher.
